# African inclusion in prostate cancer genomic studies provides the first glimpses into addressing health disparities through tailored clinical care

**DOI:** 10.1002/ctm2.1142

**Published:** 2023-01-11

**Authors:** Vanessa M. Hayes, Tingting Gong, Shingai B. A. Mutambirwa, Weerachai Jaratlerdsiri, M. S. Riana Bornman

**Affiliations:** ^1^ Ancestry and Health Genomics Laboratory, Charles Perkins Centre, School of Medical Sciences, Faculty of Medicine and Health University of Sydney Camperdown Australia; ^2^ Southern African Prostate Cancer Study, School of Health Systems and Public Health University of Pretoria Pretoria South Africa; ^3^ Department of Urology Sefako Makgatho Health Science University Ga‐Rankuwa South Africa

1

In 2010, Archbishop Emeritus Desmond Tutu became the first African to contribute his complete genome to science.^1^ His vision, African inclusion in the benefits of genomic medicine. Spending a lifetime fighting for equality, this was another battle where Africa and its peoples remain disproportionately excluded. But there was more to Tutu's vision. Over a decade earlier, at age 66 years, he had been diagnosed with advanced prostate cancer (PCa). Aware that his southern African ethnicity and genetic ancestral heritage placed him at a significant almost 3‐fold increased global risk for associated mortality,^2^ he was grateful to the team of doctors around the world who fought his battle alongside him, which he sadly lost on 26 December 2021.

While the average man from Sub‐Saharan Africa has limited to no access to current advances in PCa care, conversely, this care has almost exclusively been based on research derived from men of European ancestry. Till now, we have had no understanding of the knowledge gained from extensive PCa genome sequencing efforts, which would be of clinical relevance to men from Sub‐Saharan Africa. On 31 August 2022, we published two papers simultaneously in Nature and Genome Medicine, aimed at providing a first glimpse into the genomic contribution to PCa health disparity for men from Sub‐Saharan Africa.^3^, ^4^ Generating deep sequenced blood and matched tumour genome data for 183 largely treatment naïve and clinicopathologically aggressive disease‐presenting patients, besides 53 Australians and seven Brazilians, the study included 123 South Africans. Through ancestral interrogation of inherited variation, patients were further genetically classified as African (*n* = 113; all South African), European (*n* = 61; 53 Australian, five South African, and three Brazilian) or Admixed (*n* = 9; five South African and four Brazilian). In the Nature paper, we assessed for all types of cancer drivers, molecular subtypes, and mutational signatures, while for the Genome Medicine paper we fine‐tuned the interrogation of structural variations (SVs) which, compared to other cancer types, are more likely to be key contributors to prostate tumourigenesis. Here we provide commentary on the clinical relevance of our findings as it pertains to African ancestral PCa disparities.

Tumour mutation burden (TMB) derived from the total number of acquired single nucleotide variations and insertions/deletions (indels) < 50 bases and percentage genome alteration defined as copy number (CN) gains and losses, were significantly elevated for African versus European derived tumours, median 1.197 versus 1.061 mutations/Mb (*p* = 0.01308) and 7.26% versus 2.82% (*p* = 0.02063), respectively. While overall there was no significant difference in the burden of somatic SVs (≥50 bases), hyper‐SV tumours defined as > 100 SVs with at least 50% dominated by a single SV‐type, were notably more likely to be African derived. The significance of an elevated TMB opens two opportunities for clinical consideration. Firstly, the hope that immune checkpoint inhibitors to treat advanced PCa for men of African ancestry would be more favourable than that observed for men of European ancestry with largely mutationally ‘quiet’ tumours.^5^ Secondly, built on the observation that mutational burden is highly correlated with known carcinogenic tumour types,^6^ elevated TMB raises the potential role for an actionable mutagenic agent at play within Sub‐Saharan Africa. The latter was further supported by a significant elevation in the number, including novelty and type, of mutational signatures (caused by exogenous or endogenous exposures), observed in African over European‐derived tumours (10 vs. 1).

Using all types of somatic variation, we describe a novel PCa molecular taxonomy, defined as a clustering of tumours based on patterns of acquired genomic variation. Referred to as Global Mutational Subtypes (GMS), we identify two as African‐specific (GMS‐B and D), one as equally represented by African and European derived tumours (GMS‐C), while one appears as geo‐ancestrally universal (GMS‐A, including publicly available Asian data). Notably, a single European South African‐derived tumour presented with the mutationally ‘noisy’ otherwise African‐specific GMS‐D, which raises further speculation on the possible role of a yet unknown environmental and possibly modifiable factor contributing, at least in part, to aggressive PCa within Africa. While African‐derived tumours were presented across all the GMSs, it was notable that the universal GMS‐A defined as mutationally ‘quiet’, was the only GMS to include Asian‐derived tumours, the geo‐ancestral identifier representing the lowest incidence and mortality rates.^2^ Furthermore, in our European ancestral Australian cohort, we found GMS‐A to be associated with favourable outcomes, defined as survival or lack of biochemical relapse, compared to the CN loss predominant GMS‐C. Taken together, we speculate that African‐derived tumours represent a larger spectrum of disease heterogeneity, that includes both unfavourable and favourable outcomes.

Taking a closer look at potential oncogenic drivers, we found African tumours to present with a larger number of potentially damaging small variants (median 14 vs. 11, *p* = 0.01308), while significant ancestral‐based differences in oncogenic target genes were largely driven by CN alternations and/or SVs. The common to European *ERG* fusions, including *TMPRSS2‐ERG*, were significantly under‐represented in African tumours (37.7% *vs* 13.3%, *P* = 0.0004), while private *ERG* (12 vs. 3, *p* = 0.00035) or *TMPRSS2* fusion partners (15 vs. 4, *p* = 0.000054) were more common to Europeans. However, concurring with European‐biased data, African tumours were commonly mutated for *TP53* (52.2%), *SHBG* (49.6%), *PTEN* (38.9%), *FOXP1* (20.4%), *FOXA1* (19.5%), and *SPOP* (10.6%), and while at lower frequencies *CDK12* (6.2%) and *CSMD3* (9.7%) were 3.9 and 2‐fold more likely to be mutated in African tumours, respectively.^7^

Oncogenic genes significantly mutated in African versus European‐derived tumours included *NCOA2* (50.4% vs. 14,8%, *p* < 0.0001) and *PCAT1* (13.3% vs. 1.6%, *p* = 0.0117), and new to PCa *SETBP1* (32.7% vs. 14.8%, *p* = 0.0115), *STK19* (24.8% vs. 6.6%, *p* = 0.0035) and not quite significant *RABGAP1L* (9.7% vs. 1.6%, *p* = 0.0587). African predominant cancer drivers at frequencies ≥10% include *EPHA6* (3.5‐fold frequency increase in Africans), *CADM2* (1.9)*, PDE4D* (1.8)*, LRP1B* (1.5) and *PTPRD* (1.4) and new to PCa *TYW1* (2.1), *BRAF* (2.0), and *MACROD2* (1.4). A detailed summary of potential clinical implications for African significant and predominant cancer drivers can be found in Table 1. While we have previously shown that men from Southern Africa are at 2.1‐fold greater risk for advanced PCa compared with African Americans,^8^ it was notable that *EPHA6*, while new to PCa, has been shown to be recurrently mutated in colon cancers from African Americans.^9^ Excluding ancestry‐specific *ERG/TMPRSS2* fusion partners, cancer drivers uniquely mutated in Africans included, *MFF* (eight tumours), *FAT4* (7), *HTRA3* (7), *MC2L2* (6), *CHD3* (5), *MUC17* (5), and *TEC* (2), and in Europeans *MTCH2* (4) and *PAPSS2* (3), representing a longer tail of actionable therapeutic targets for Africans.

**TABLE 1:** Clinical implications for African significant and predominant prostate cancer driver genes

Gene	Evidence for clinical implications	References^1^
*NCOA2*	Prognostic marker for aggressive PCa disease and poor outcomes for mCRPCa.	Silva et al. Genes Chromosomes Cancer. 2016; Fettke et al. Prostate. 2021
*PCAT1*	Prognosis PCa and long non‐coding RNA‐based putative therapeutic target.	Ghafouri‐Fard et al. Exp Mol Pathol. 2020; An et al. Am J Clin Exp Urol. 2022
*SETBP1*	**New to PCa**. Associated with poor outcomes in myeloid leukaemias.	Patnaik et al. Hematology Am Soc Hematol Educ Program. 2020
*STK19*	**New to PCa**. Serine/threonine‐protein kinase‐based inhibition therapeutic potential, although its true oncogenic function is under question.	Asquith et al. Nat Rev Drug Discov. 2020; Rodríguez‐Martínez et al. Cell. 2020
*RABGAP1L*	**New to PCa**. Chromosomal imbalances in nervous system tumours, fusions in liver cancer.	Ross et al. Oncologist. 2014; Asai et al. Gene. 2015
*EPHA6*	Overexpressed in metastatic PCa and proposed therapeutic target.	Li et al. Oncotarget. 2015
*CADM2*	PCa tumour suppressor reduces colon malignancy when upregulated.	Chang et al. Clin Cancer Res. 2010; Wang et al. Mol Biotechnol. 2022
*PDE4D*	The target for small molecule inhibition is capable of overcoming PCa chemoresistance.	Powers et al. Mol Cancer Res. 2015; Xie et al. Biomedicines. 2021
*LRP1B*	Mutated form improves outcomes with immune checkpoint inhibitors, while up‐regulation reduces hypoxia‐driven prostate tumourigenesis.	Zheng et al. Exp Mol Pathol. 2019; Brown et al. J Immunother Cancer. 2021
*PTPRD*	Mutated at low frequencies in PCa. Prognostic in various cancers, a mutated form associated with higher TMB scores and better overall survival with immune checkpoint inhibitors.	Nunes‐Xavier et al. Molecular Cell Research. 2019; Ou et al. Math Biosci Eng. 2022
*TYW1*	**New to PCa**. Fusions in leukaemia.	Panagopoulos et al. Exp Hematol Oncol. 2019
*MACROD2*	**New to PCa**. Driver mutations in colon, liver and breast cancer. Expression predicts response to chemotherapy. Loss causes repression of PARP1 activity, impairing DNA repair.	Fujimoto et al. Nat Genet. 2016; van den Broek et al. Oncotarget. 2018; Sakthianandeswaren et al. Cancer Discov. 2018; Feijs et al. Cancers. 2020
*BRAF*	**New to PCa**. Activating mutations in multiple cancer types. Serine/threonine kinase druggable target and therapy response.	Davies et al. Nature. 2002; Dankner et al. Oncogene. 2018

^1^ Full list of supporting references can be found on the Hayes Lab webpage (www.hayeslab.net).

In conclusion, the significant differences in oncogenic drivers and mutational patterns observed for our African cohort, suggest that current European‐biased genomic PCa resources are limited in their applicability to provide optimal African‐relevant PCa care. As such, we strongly advocate for further inclusion of diverse populations across the ancestral spectrum of Sub‐Saharan Africa in PCa genomic studies and further African‐specific validation for potential clinical relevance.

## CONFLICT OF INTEREST

The authors declare that they have no conflict of interest.
